# 428. Risk Stratification and Clinical Outcomes in a Mixed Vaccination Cohort of COVID-19 Patients Admitted in a Tertiary Hospital Using ISARIC 4C Scores

**DOI:** 10.1093/ofid/ofad500.498

**Published:** 2023-11-27

**Authors:** May Anne S Pascual, John Robinson L Reyes

**Affiliations:** Dr Paulino J Garcia Memorial Research and Medical Center, cabanatuan city, Nueva Ecija, Philippines; Dr Paulino J Garcia Memorial Research and Medical Center, cabanatuan city, Nueva Ecija, Philippines

## Abstract

**Background:**

The risk of clinical deterioration and death with the use of prognostic tools remain necessary for strategic planning among COVID-19 patients. The International Severe Acute Respiratory and Emerging Infections Consortium (ISARIC) developed and validated Coronavirus Clinical Characterization Consortium (4C) scores to predict clinical deterioration and mortality. ISARIC 4C scores uses readily available clinical parameters. With the increasing uptake of COVID-19 vaccine, there is a need to evaluate these tools. The study examined a cohort of fully vaccinated (FV), partially vaccinated (PV) and unvaccinated (UV) COVID-19 patients and determined their clinical outcomes and stratified risks using ISARIC 4C scores.

**Methods:**

We reviewed medical records of confirmed COVID-19 patients admitted to a single tertiary hospital between May 2021 and April 2022. Vaccination status were classified as FV, PV and UV. The primary outcome was in-hospital mortality. Baseline data were collected and predictors recorded upon admission were used to calculate the 4C scores. Multiple logistic regression analysis was used to determine the association of vaccination status and 4C Deterioration and Mortality scores with in-hospital mortality. STATA 14 was used for data analysis and p< 0.05 was considered statistically significant.

**Results:**

A total of 301 confirmed COVID-19 patients were included and 46.5% were fully vaccinated. FV patients had significantly lower CRP (p< 0.0001), had higher mean oxygen saturation (p< 0.0001), had lower mean mortality score (p=0.004), had lower mean % risk for deterioration (p< 0.0001) and had significantly lower 30-day mortality (< 0.0001) compared to those UV and PV. Patient who died had significantly higher mean age (p=0.001), lower mean lymphocyte ratio (p=0.0004), had significantly higher mean deterioration and mortality score (p< 0.0001). Being fully vaccinated was associated with lower risk for mortality (OR=0.4, 95%CI=0.2 to 0.7).

**Conclusion:**

Fully vaccinated patients were at lower risk for deterioration and mortality. High ISARIC 4C scores were also significantly associated with higher risk for mortality. Risk stratification tools should be utilized in COVID-19 management as they provide a helpful means to identify patients at risk for worse outcomes.

**Disclosures:**

**All Authors**: No reported disclosures

